# *Pseudomonas aeruginosa* Planktonic- and Biofilm-Conditioned Media Elicit Discrete Metabolic Responses in Human Macrophages

**DOI:** 10.3390/cells9102260

**Published:** 2020-10-09

**Authors:** Amanda L. Fuchs, Isaac R. Miller, Sage M. Schiller, Mary Cloud B. Ammons, Brian Eilers, Brian Tripet, Valérie Copié

**Affiliations:** Department of Chemistry and Biochemistry, Montana State University, Bozeman, MT 59717, USA

**Keywords:** *Pseudomonas aeruginosa*, biofilm, bacterial-conditioned media, NMR metabolomics, human macrophages, immunometabolism

## Abstract

Macrophages (MΦs) are prevalent innate immune cells, present throughout human bodily tissues where they orchestrate innate and adaptive immune responses to maintain cellular homeostasis. MΦs have the capacity to display a wide array of functional phenotypes due to different microenvironmental cues, particularly soluble bacterial secretory products. Recent evidence has emerged demonstrating that metabolism supports MΦ function and plasticity, in addition to energy and biomolecular precursor production. In this study, 1D ^1^H-NMR-based metabolomics was used to identify the metabolic pathways that are differentially altered following primary human monocyte-derived MΦ exposure to *P. aeruginosa* planktonic- and biofilm-conditioned media (PCM and BCM). Metabolic profiling of PCM- and BCM-exposed MΦs indicated a significant increase in glycolytic metabolism, purine biosynthesis, and inositol phosphate metabolism. In addition, these metabolic patterns suggested that BCM-exposed MΦs exhibit a hyperinflammatory metabolic profile with reduced glycerol metabolism and elevated catabolism of lactate and amino acids, relative to PCM-exposed MΦs. Altogether, our study reveals novel findings concerning the metabolic modulation of human MΦs after exposure to secretory microbial products and contributes additional knowledge to the field of immunometabolism in MΦs.

## 1. Introduction

The host innate immune system provides the initial line of defense against invasive microbes and infection [1]. Macrophages (MΦs), a type of phagocytic innate immune cell, are crucial for host defense processes, including pathogen and infection clearance [2], and substantial evidence supports that MΦs are key regulators of wound healing [3,4]. Bacterial infection induces the migration and activation of immune cells, including neutrophils and monocytes, as a result of cytokine and chemokine secretion at the site of infection [5]. In acute wounds, MΦs phagocytose debris, bacteria, and apoptotic host cells from the wound bed as part of an inflammatory response, prior to the initiation of a proliferative phase and tissue regeneration, which is marked by a phenotypic transition of MΦs from pro-inflammatory, M1 MΦs, to anti-inflammatory, M2 MΦs [6,7]. Progression from inflammation to the proliferative and remodeling phases of healing does not readily occur in chronic wounds, which leads to a prolonged presence of M1 MΦs in the chronic wound bed and delayed tissue regeneration [8,9]. However, the detailed mechanisms by which this phenomenon takes place remain poorly understood and are the focus of numerous research studies. Novel insight gained from investigating the molecular events and biological processes that support persistent inflammation in chronic wounds has the potential to provide valuable knowledges to guide the development of more effective therapeutic treatments against biofilm infections in chronic wounds.

Biofilms are structured colonies of bacterial cells encased in a secreted extracellular polymeric matrix, which adhere to biological or abiotic surfaces [10,11]. Bacteria within biofilms are phenotypically distinct from planktonic, or free-living, bacteria and are inherently more resistant to antibiotics and host defense mechanisms [12,13]. Chronic wounds are particularly susceptible to bacterial infection and subsequent biofilm colonization of the wound bed due to inadequate blood flow, hypoxia [14], delayed re-epithelialization [15], and impaired host defense mechanisms [16,17]. A previous study demonstrated that bacteria present within chronic wounds grow predominantly as biofilms, whereas acute wounds exhibit minimal biofilm content [18]. Furthermore, partial thickness porcine wound models have shown that wounds inoculated with a wound isolate bacterial strain display biofilm structure formation within 48 h [19]. Moreover, biofilm-challenged diabetic murine punch biopsy wounds have been shown to exhibit delayed healing, prolonged inflammation, and extensive tissue necrosis compared to control wounds [20], suggesting that biofilm formation within wounds contributes to deferred healing and wound chronicity. Although biofilm colonization within chronic wounds is well established, interactions of bacterial biofilms with host innate immune cells, which may support pathogenesis, remain largely uncharacterized.

While chronic wound biofilms are known to maintain a diverse flora of microbes, the most prevalent bacteria in diabetic foot, venous leg, and pressure ulcers include *Staphylococcus*, *Pseudomonas*, *Peptoniphilus*, *Enterobacter*, *Stenotrophomonas*, *Finegoldia*, and *Serratia* spp [21]. In addition, chronic wound microbiota vary markedly between different wound types and individuals, with most exhibiting colonization by multiple microbial species [21]. This complexity contributes to and often confounds the determination of a definitive host immune response. Consequently, and in order to further understand and gain insight into the role of biofilms in pathogenesis, a single opportunistic pathogen, *Pseudomonas aeruginosa*, was examined in this study. *P. aeruginosa* is a particularly virulent pathogen that is found predominantly in venous leg ulcers [21]. Moreover, chronic *P. aeruginosa* infections are correlated with increased morbidity, mortality, and worsened disease progression [22,23]. Previous studies demonstrated that biofilm-conditioned media (BCM), consisting of spent growth medium and soluble, secreted bacterial products, induces distinct morphological changes, diminished viability, apoptosis, and altered cytokine production in keratinocytes and fibroblasts, when compared to these cell types exposed to planktonic-conditioned media (PCM) [24,25].

Notably, growing evidence has indicated that immune cell phenotypes and responses to environmental clues, including pathogen exposure, are reflected in characteristic metabolic changes and adaptations, also known as metabotypes [26]. These recent discoveries have been contributing to the rapidly developing and expanding field of immunometabolism, a field at the forefront of immunology that is focused on understanding the regulatory networks underlying the crosstalk between immune cell function and metabolism [27,28]. The relatively new field of immunometabolism bridges biochemical and immunological research and has been enhanced by significant technological advances in metabolomics [29]. The latter involves the global profiling of metabolite levels using platforms, such as mass spectrometry and NMR [30,31], and aims to investigate the wide-ranging metabolic changes and differential modulation of metabolic pathways occurring in biological samples, such as cells, tissues, and biofluids, by identifying and quantifying perturbations in metabolite profiles [32,33].

Recent studies have demonstrated that metabolites, such as itaconate and succinate, can serve as signaling molecules, in addition to being metabolic intermediates, and mediate the resultant functional phenotypes of MΦs [34,35]. Prior research has shown that M1 and M2 MΦs exhibit distinct metabolite profiles involving central metabolism, including oxidative phosphorylation, fatty acid utilization, and glycolysis. It is therefore thought that the immunometabolic regulatory axes associated with immune cell activation are vital for their functionality [36,37,38]. However, further research is needed to better understand the immunometabolic characteristics of human MΦs and to elucidate the potential metabolic and functional differences associated with host MΦ responses to invasive pathogens, with specific emphasis placed on the differential phenotypic responses of MΦs when challenged with planktonic versus biofilm bacteria, and their respective secreted bacterial products.

In the present study, we aimed to investigate the metabolic impact of *P. aeruginosa* PCM and BCM exposure on primary human monocyte-derived resting (M0) MΦs, using CD14^+^ magnetic-activated cell sorting (MACS), in vitro MΦ differentiation and exposure schemes, 1D ^1^H NMR metabolomics, metabolic profiling using Chenomx Suite software, and statistical analysis. M0 MΦs were differentiated from primary human monocytes using MΦ colony-stimulating factor (M-CSF) for 9 days, prior to stimulation with PCM or BCM. Following exposure, both MΦ intra- and extracellular metabolites were extracted, 1D ^1^H NMR spectra were recorded, and metabolite profiling of resulting metabolite extract-derived NMR spectra was conducted. Results from this study highlight significant changes in metabolite profiles and reveal several metabolic pathways that are differentially modulated in PCM- and BCM-exposed MΦs, including glycolysis, purine biosynthesis, inositol phosphate metabolism, branched-chain amino acid catabolism, and glycerol metabolism. The functional significance of the distinct metabolic profiles found to be associated with PCM- and BCM-exposed MΦ phenotypes are also reviewed.

## 2. Materials and Methods

### 2.1. Isolation of Primary Human Monocytes

Heparinized whole blood was collected in compliance with proper, ethical guidelines, Montana State University’s Institutional Review Board approval (ID# 00000799; Protocol #VC100118), and signed, informed consent from healthy, adult blood donor volunteers in Bozeman, Montana, USA. A total of 5 donors that ranged in age from 19 to 35 were included in this particular study, of whom 40% of these volunteers were female. Peripheral blood mononuclear cells were isolated by differential centrifugation in lymphocyte separation (Ficoll) media (Corning Inc., Corning, NY, USA) at 800× g for 25 min at room temperature, following protocols described previously [39]. CD14^+^ monocytes were isolated from peripheral blood mononuclear cells by MACS using CD14 human microbeads (Miltenyi Biotec, San Diego, CA, USA), which amounted to an average purity of 97.1 ± 1.3% (Supplementary Materials, Figure S1) when analyzed on an LSR Fortessa flow cytometer (BD Biosciences, San Jose, CA, USA).

### 2.2. In vitro Differentiation of Primary Human Monocyte-Derived MΦs

To obtain primary human monocyte-derived MΦs, CD14^+^ monocytes were seeded and cultured at a concentration of 1 × 10^6^ cells per mL in 25 cm^2^ tissue culture flasks (Corning Inc., Corning, NY, USA) with RPMI 1640 media containing l-glutamine (Lonza, Morristown, NJ, USA), supplemented with sodium pyruvate (1 mM; Lonza, Morristown, NJ, USA), 1X non-essential amino acids (NEAAs; Gibco, ThermoFisher Scientific, Waltham, MA, USA), 10% (*v*/*v*) fetal bovine serum (FBS; ATCC, Manassas, VA, USA), and 50 ng/mL recombinant human M-CSF (PeproTech Inc., Cranbury, NJ, USA) for 9 days at 37 °C, 5% CO_2_. Media and cytokines were replenished every 3 days. Following differentiation, cell surface marker characterization of primary human monocyte-derived MΦs was performed using flow cytometry (Figure S2).

### 2.3. Fluorescent-Conjugated Antibody Staining and Flow Cytometry Analysis

Primary human monocytes isolated using CD14^+^ MACS were stained with anti-human antibodies directed against CD14 (BD Biosciences, San Jose, CA, USA) and corresponding isotype controls. M0 MΦs were stained with anti-human antibodies directed against CD68, CD80, and CD163 (BD Biosciences, San Jose, CA, USA) and corresponding isotype controls. Cellular permeabilization was conducted on a subset of cells using Cytofix/Cytoperm (BD Biosciences, San Jose, CA, USA) for CD68 intracellular staining. After antibody staining, cells were reconstituted in fluorescence-activated cell sorting (FACS) buffer for subsequent analysis on an LSR Fortessa flow cytometer (BD Biosciences, San Jose, CA, USA). Analysis of FACS data was conducted using FCSalyzer software (version 0.9.14-alpha) with both monocytes and MΦs gated based upon cellular size and singlet cells. Since fluorescence levels at baseline differed between our different fluorophores, mean fluorescence intensity (MFI) values were normalized to their corresponding isotype control MFI (normalized MFI; Supplementary Figure S2).

### 2.4. Biofilm-Conditioned Medium (BCM)

BCM was generated using methods similar to those described previously [24,25]. In brief, 6-well plate tissue culture inserts (0.4 µm; Falcon) were inoculated with five 10 µL droplets of overnight *P. aeruginosa* PAO1 culture grown in 10% (*v*/*v*) Brain Heart Infusion (BHI; BD Biosciences, San Jose, CA, USA) at 37 °C and 220 rpm agitation. Following inoculation, tissue culture inserts were placed into a 6-well tissue culture plate, and 1.5 mL of 10% (*v*/*v*) BHI was added to each well. The tissue culture plate was incubated at 37 °C, and media were refreshed every 24 h to maintain biofilm viability [40]. After 72 h of growth, biofilm-containing inserts were transferred to a new 6-well tissue culture plate, 1.5 mL of 1X phosphate buffered saline (PBS) was added to each well, and biofilms were incubated at 37 °C for 30 min to remove excess growth media. These mature, biofilm-containing inserts were then transferred to a new 6-well tissue culture plate, 1.5 mL of MΦ culture medium, consisting of RPMI 1640 media containing l-glutamine, supplemented with sodium pyruvate (1 mM), 1X NEAAs, and 10% FBS, was added to each well, and biofilms were incubated at 37 °C. BCM was harvested after 24 h of conditioning, centrifuged at 5000 rpm for 10 min at room temperature to remove cells, sterile-filtered (0.22 µm; ThermoFisher Scientific, Waltham, MA, USA), and stored at −20 °C. Tissue culture wells were replenished with fresh cell culture media immediately following BCM harvest. A total of five 24 h BCM collections were pooled, pH-adjusted to 7.4 using sterile, concentrated HCl or NaOH, and stored at −20 °C until further use.

Colony-forming units (CFUs) for *P. aeruginosa* PAO1 biofilms were determined after initial maturation (72 h) using 10% (*v*/*v*) BHI and following BCM generation using a serial dilution drop-plating method. In brief, biofilm colonies were removed from tissue culture inserts using 1X PBS, vortexed, agitated in a sonicator bath, serially diluted with PBS, plated on 100% tryptic soy agar plates, and incubated at 37 °C. Bacterial colonies were then counted, and the number of CFUs per insert was calculated.

### 2.5. Planktonic-Conditioned Medium (PCM)

PCM was generated to yield a similar proportion of CFUs per unit of fluid volume to that of BCM, as previously described [24,25]. In brief, an overnight culture of *P. aeruginosa* PAO1 was grown in 10% (*v*/*v*) BHI at 37 °C with 220 rpm agitation. This overnight culture was then centrifuged at 5000 rpm for 5 min at room temperature, and then the cells were washed using 1X PBS to remove excess media. This cell suspension was centrifuged again at 5000 rpm for 5 min at room temperature, and the cells were resuspended in MΦ culture medium, consisting of RPMI 1640 media containing l-glutamine, supplemented with sodium pyruvate (1 mM), 1X NEAAs, and 10% FBS, at a density equivalent to that of a mature PAO1 biofilm (6 × 10^9^ CFUs/mL). This PAO1 suspension was cultured at 37 °C with 220 rpm agitation for 24 h. After the conditioning period, PCM was centrifuged at 5000 rpm for 10 min at room temperature to remove cells, sterile-filtered (0.22 µm; ThermoFisher Scientific, Waltham, MA, USA), pH-adjusted to 7.4 using sterile, concentrated HCl or NaOH, and stored at −20 °C until further use.

### 2.6. Exposure of Primary Human Monocyte-Derived MΦs to PCM, BCM, and Control Media

Before exposure experiments, equal volumes of PCM, BCM, and 1X PBS were mixed with equal volumes of MΦ cell culture (control) media (A in Figure S3), consisting of RPMI 1640 media containing l-glutamine, supplemented with sodium pyruvate (1 mM), 1X NEAAs, and 10% FBS, and pre-warmed to 37 °C. Control media consisting of 50% (*v*/*v*) MΦ cell culture media and 50% (*v*/*v*) 1X PBS were determined to be the most appropriate exposure media for control MΦ experiments due to its metabolic similarity to PCM and BCM compared to full strength (100% *v*/*v*) MΦ cell culture media (Figure S4 and Table S1). Spent media were removed from 9 days in vitro M0 MΦ culture flasks and discarded. Adherent cells were then washed using 4 mL of pre-warmed (37 °C) Dulbecco’s PBS; this wash was then discarded. Pre-warmed 50% (*v*/*v*) PCM, BCM, and control media were transferred into 25 cm^2^ tissue culture flasks with (experimental flasks) and without (sham media control flasks) M0 MΦs (B in Figure S3), and these flasks were then all placed into a 37 °C, 5% CO_2_ incubator for 1 h before conducting cell/media harvests and intra-/extracellular metabolite extractions.

### 2.7. Intra- and Extracellular Metabolite Extraction

Following exposure, spent media (from experimental flasks; B in Supplementary Figure S3) were transferred into clean 15 mL conical tubes and centrifuged at 2000× g for 1 min at room temperature to pellet insoluble debris. Two separate 1.5 mL aliquots of sham (from sham media control flasks; B in Figure S3) and spent cell culture media were transferred to clean 1.5 mL microtubes and stored at −80 °C before extraction of extracellular metabolites. Cellular monolayers were washed using 1 mL of cold (4 °C) sterile 1X PBS. These wash solutions were transferred to a clean 15 mL conical tube, centrifuged at 2000× *g* for 1 min at room temperature to pellet non-adherent cells, decanted, and then resuspended in 0.5 mL of −20 °C 50% (*v*/*v*) aqueous methanol. An amount of 1.5 mL of −20 °C 50% (*v*/*v*) aqueous methanol was pipetted into 25 cm^2^ tissue culture flasks in order to synchronously quench, extract, and detach cells from the surface of the flask with the aid of a cell scraper. Cell suspensions were then transferred to clean 15 mL conical tubes, which already contained non-adherent cell suspensions, and thoroughly mixed.

Two aliquots of this cell suspension (1 mL each) were transferred into separate, clean 2 mL lysis B matrix tubes (MP Biomedicals, Santa Ana, CA, USA) and lysed using 2 cycles (40 s each) on a FastPrep-24 5G homogenizer (MP Biomedicals, Santa Ana, CA, USA) at a speed of 6.0 m/second, with lysis tubes being stored on ice in between cycles. A 50 µL aliquot of cell lysate was stored at −80 °C for subsequent determination of protein content, and then 0.5 mL of chloroform was added to each cell lysis tube. Tubes were vortexed for 3 consecutive cycles of 10 s each, and then incubated at −20 °C for 20 min, before centrifugation at 10,000× *g* for 10 min at room temperature to separate aqueous and nonpolar phases [41]. The aqueous phase was transferred into a 1.5 mL microtube, dried overnight using a vacuum centrifuge (Speedvac) with no heat, and stored at −80 °C.

Sham and spent cell culture media aliquots were filtered using 3 kDa molecular weight cutoff centrifugal filters (MilliporeSigma, Burlington, MA, USA), which had been prewashed extensively as previously described [40], before being dried overnight using a vacuum centrifuge (Speedvac) with no heat and stored at −80 °C.

### 2.8. Protein Assay

Sample protein content was established using a BCA (bicinchoninic acid) protein assay (ThermoFisher Scientific, Waltham, MA, USA; Cat. No. 23225) and used to normalize intra- and extracellular metabolite concentrations.

### 2.9. NMR Sample Preparation

Intra- and extracellular metabolite extracts were reconstituted in 0.6 mL of NMR buffer, which consisted of 25 mM NaH_2_PO_4_/Na_2_HPO_4_, 0.4 mM imidazole, 0.25 mM 4,4-dimethyl-4-silapentane-1-sulfonic acid (DSS) in 90% (*v*/*v*) H_2_O/10% (*v*/*v*) D_2_O at pH 7.0. Samples were subjected to centrifugation at 21,000 rpm for 60 s at room temperature to pellet insoluble material, and then transferred into 5 mm NMR tubes for subsequent metabolomics analysis.

### 2.10. NMR Data Acquisition and Preprocessing

NMR spectra were collected at 25 °C (298 K) on a Bruker 600 MHz (^1^H Larmor frequency) AVANCE III solution NMR spectrometer, equipped with an automatic sample loading system (SampleJet), a 5 mm triple resonance (^1^H, ^15^N, ^13^C), three channel inverse, liquid-helium-cooled NMR cryoprobe, and Topspin software (Bruker Scientific LLC, Billerica, MA, USA; version 3.6). 1D ^1^H-NMR spectral acquisitions were performed using the Bruker-supplied ‘zgesgp’ pulse sequence [42,43]; NMR spectra were acquired with 256 scans and a 9615.38 Hz ^1^H spectral window. Free induction decays were recorded with 32,000 data points and a dwell time interval of 52 microseconds, equating to a total data acquisition time of 1.7 s. Recovery delay times between data acquisitions were fixed at 1 s, amounting to an overall 2.7 s relaxation recovery delay in between scans [44,45]. Spectral phase correction and chemical shift referencing of DSS were performed using Topspin software (Bruker Scientific LLC, Billerica, MA, USA; version 3.6). Metabolite assignments, annotated using Chenomx NMR Suite software (version 8.1; Chenomx Inc., Edmonton, Alberta, Canada) as detailed in the “NMR Data Analysis” section below, were further validated using 2D ^1^H-^1^H total correlation spectroscopy (TOCSY) NMR as previously described [39] or by spiking metabolite reference standards into the samples, as needed.

### 2.11. Analysis of NMR Data

Additional processing of ^1^H NMR spectra and metabolic profiling were performed using Chenomx. Preprocessed ‘1r’ NMR spectra files were imported into Chenomx and baseline correction was conducted using the automatic cubic spline function followed by manual breakpoint adjustment to procure a flat and well-defined baseline [46,47]. All ^1^H chemical shifts were referenced to the 0.0 ppm DSS signal, and imidazole ^1^H-NMR signals were utilized to make slight chemical shift adjustments arising from minor sample pH variation. Identification and quantitation of metabolites was performed by manually fitting 1D ^1^H spectral peak-based patterns, intensities, and chemical shifts with reference to small molecule spectral patterns present in the Chenomx database for 600 MHz (^1^H Larmor frequency) NMR spectrometers; manual adjustments were performed to attain optimal spectral pattern fits based upon metabolite intensity and peak cluster location [48]. In addition to serving as a chemical shift reference, the internal DSS (0.25 mM) standard was also used for metabolite quantitation. Metabolite concentrations determined for spent media (from experimental flasks; B in Figure S3) metabolite extracts were normalized using metabolite concentrations established for sham media (from sham media control flasks; B in Figure S3) to accurately depict concentration changes, metabolite consumption (negative values) and/or secretion (positive values), arising directly from 1 h exposure of MΦs to PCM, BCM, and control media (C in Figure S3).

### 2.12. Statistical Analysis

Metabolite concentrations were normalized to protein content and NMR buffer volume before multivariate statistical analysis using MetaboAnalyst 4.0 [49,50]. After normalization, metabolite concentrations were log-transformed, auto-scaled, mean-centered, and divided by standard deviation, to yield a Gaussian data distribution prior to multivariate statistical analysis, which included 2D-principal component analysis (PCA) and hierarchical clustering analysis (HCA). A Euclidean distance measure and Ward clustering algorithm were used when performing HCA in MetaboAnalyst. GraphPad Prism program version 8.2.1 (GraphPad Software, San Diego, CA, USA) was used to establish statistical significance using an unpaired parametric two-tailed *t*-test with Welch’s correction.

## 3. Results

### 3.1. NMR Analysis of Metabolite Extracts Derived from MΦs Exposed to PCM, BCM, or Control Media Reveals Spectral Pattern Differences

**Figure 1:**
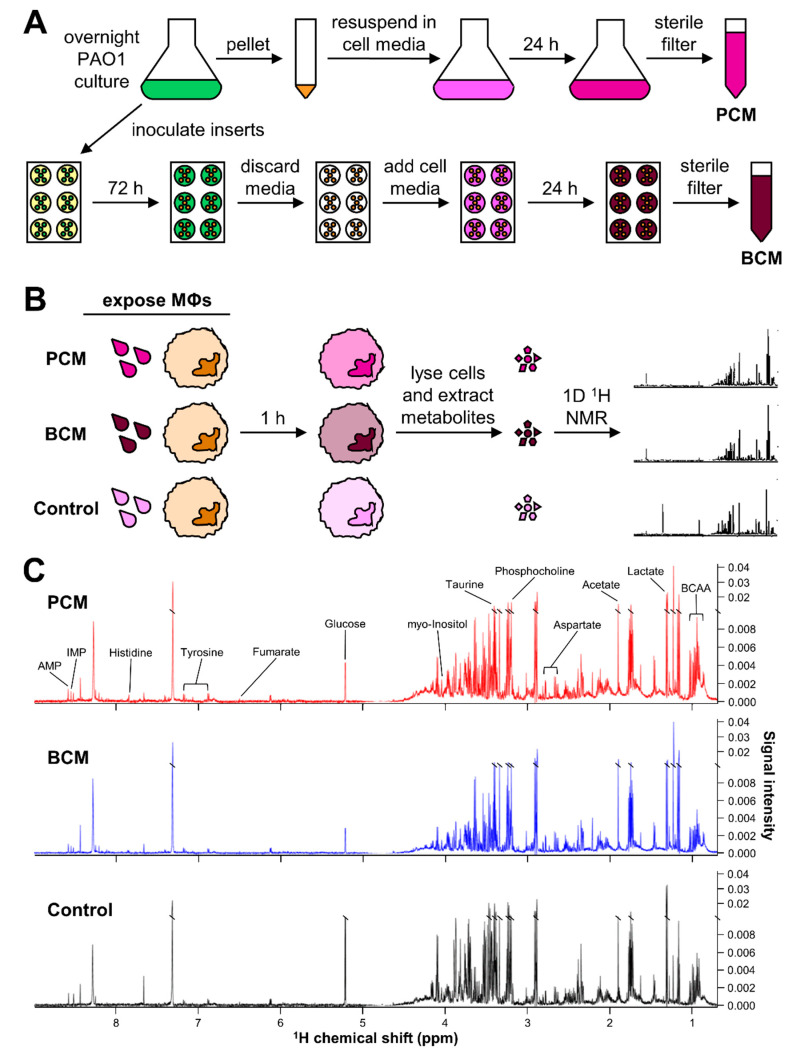
Overview of experimental design and 1D ^1^H-NMR methods. Schematic overview of experimental approaches concerning (**A**) the generation of planktonic-conditioned media (PCM) and biofilm-conditioned media (BCM), and (**B**) the exposure of primary human monocyte-derived macrophages (MΦs) to PCM, BCM, and control media followed by cell lysis, metabolite extraction, and 1D ^1^H-NMR analysis. (**C**) Representative 1D ^1^H-NMR spectra of intracellular metabolite extracts derived from primary human monocyte-derived MΦs exposed to PCM (red), BCM (blue), and control (black) media acquired on a Bruker 600 MHz (^1^H Larmor frequency) NMR spectrometer at Montana State University. Visually distinct metabolite signals are indicated above the NMR spectra. Abbreviations denote: AMP, adenosine monophosphate; BCAA, branched-chain amino acids; IMP, inosine monophosphate.

To assess the metabolic impact of *P. aeruginosa* PCM and BCM exposure on primary human MΦs, it was imperative to first generate *P. aeruginosa* PCM and BCM. For this purpose, *P. aeruginosa* biofilms were grown on 6-well tissue culture inserts and used to produce BCM. The initial cell density of each insert immediately following inoculation was 6.50 ± 1.22 × 10^7^ CFUs/insert, and after 72 h of growth in 10% (*v*/*v*) BHI the biofilms had reached a cell density of 2.10 ± 0.51 × 10^9^ CFUs/insert (Figure S5). These mature biofilm inserts were then incubated with 1.50 mL of cell culture medium for 24 h to generate BCM, resulting in a final cell density of 9.87 ± 1.10 × 10^9^ CFUs/mL (Figure 1A). The pH of the BCM was 8.43 ± 0.17 after the conditioning period, and it was adjusted to 7.4 prior to sterile filtration and storage at −20 °C.

Planktonic *P. aeruginosa* was grown in 10% (*v*/*v*) BHI for 16 h at 37 °C with 220 rpm agitation, resulting in a cell density of 2.94 ± 0.89 × 10^9^ CFUs/mL. These planktonic cells were then centrifuged, washed, resuspended in cell culture media, and incubated for 24 h at 37 °C with 220 rpm agitation, resulting in a final cell density of 1.04 ± 0.49 × 10^10^ CFUs/mL (Figure 1A). This PCM had a slightly higher cell density than the BCM; therefore, prior to experimental use, PCM volume was adjusted to be equivalent to the CFUs/mL of the BCM. Thus, primary human MΦs exposed to PCM and BCM were in contact with medium that had been conditioned by approximately 9.9 × 10^9^ CFUs/mL. The pH of the PCM was measured to be 7.60 ± 0.24 after the conditioning period, and it was adjusted to 7.4 prior to sterile filtration and storage at −20 °C.

Anticipating the potential influence of *P. aeruginosa* PCM and BCM on primary human MΦ metabolism, parallel intra- and extracellular 1D ^1^H-NMR metabolomics analyses were performed on PCM-exposed, BCM-exposed, and control MΦs, as outlined in Figure 1B. Intra- and extracellular metabolites were extracted from primary human MΦ cultures after 1 h of exposure to PCM, BCM, or control media conditions. Metabolite extracts obtained from PCM-exposed, BCM-exposed, and control primary human MΦs (Figure 1B) were analyzed using a Bruker 600 MHz (^1^H Larmor frequency) NMR spectrometer, and corresponding 1D ^1^H NMR spectra were processed and profiled using Topspin and Chenomx NMR Suite software, respectively. Representative 1D ^1^H NMR spectra acquired from these metabolite extracts revealed clear signal intensity differences and distinct metabolic profiles between PCM-exposed, BCM-exposed, and control primary human MΦs (Figure 1C; Figure S6). A total of 48 metabolites were identified across all samples, with coverage of 15 different metabolite subclasses (Tables S2 and S3). Over half of the identified metabolites belonged to the carboxylic acids and derivatives class (28 unique metabolites), with the majority of these species being affiliated with the amino acids, peptides, and analogues subclass (23 unique metabolites) (Table S2). Approximately 10.4% of the total metabolite diversity was attributable to the organooxygen compounds class, with five unique metabolite species (Table S2). Metabolites identified within the purine nucleotides class, which comprised 8.3% of the metabolites detected, included adenosine diphosphate (ADP), adenosine monophosphate (AMP), guanosine triphosphate (GTP), and inosine monophosphate (IMP) (Tables S2 and S3).

### 3.2. Multivariate Statistical Analysis of Quantitative Metabolic Data Identifies Metabolic Differences between MΦ Exposure Groups

**Figure 2:**
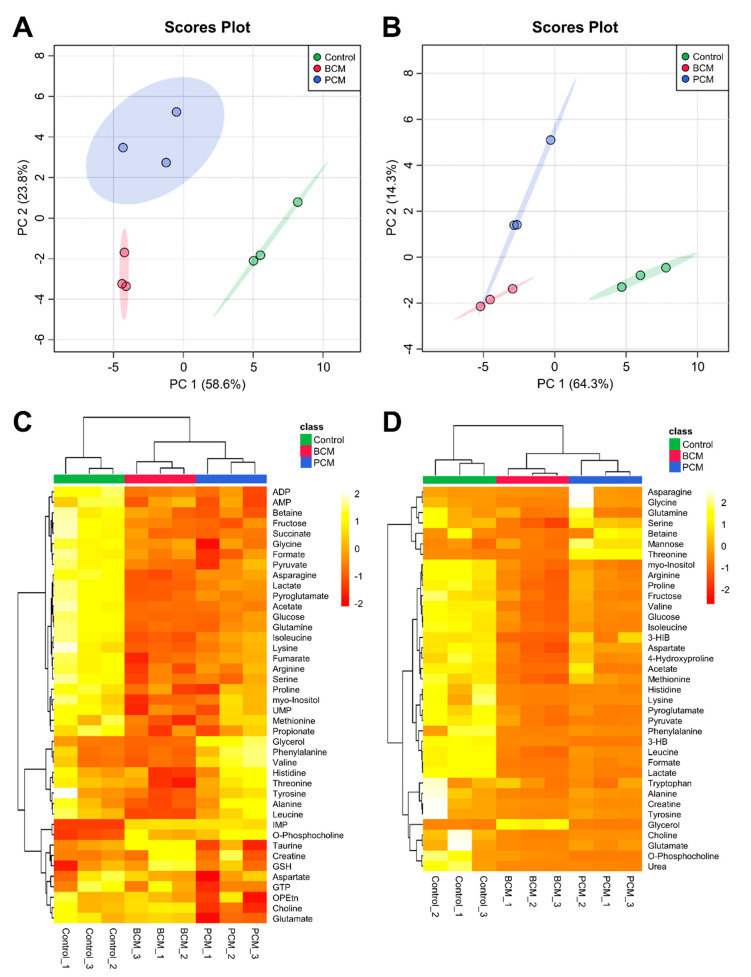
Multivariate statistical analysis of intra- and extracellular metabolomes derived from MΦs exposed to PCM, BCM, and control media. Two-dimensional principal component analysis (2D-PCA) scores plots generated by analysis of metabolic profiles from (**A**) intra- and (**B**) extracellular MΦ metabolite extracts (control, green; BCM-exposed, red; PCM-exposed, blue), with shaded regions illustrating respective 95% confidence intervals. Heat map visualization and hierarchical clustering analysis (HCA) of metabolic profiles from (**C**) intra- and (**D**) extracellular MΦ metabolite extracts were conducted using a Euclidean distance measure calculated from metabolite abundance and a Ward clustering algorithm. The top column bar is colored according to experimental MΦ group (control, green; BCM-exposed, red; PCM-exposed, blue), and the color scale represents the scaled abundance of each metabolite, with pale yellow indicating high abundance and deep red indicating low abundance. Abbreviations denote: 3-HB, 3-hydroxybutyrate; 3-HIB, 3-hydroxyisobutyrate; ADP, adenosine diphosphate; AMP, adenosine monophosphate; BCM, biofilm-conditioned media; GSH, reduced glutathione; GTP, guanosine triphosphate; IMP, inosine monophosphate; OPEtn, o-phosphoethanolamine; PCM, planktonic-conditioned media; UMP, uridine monophosphate.

To examine and compare the metabolic signatures of human MΦs exposed to PCM, BCM, and control media in greater depth, multivariate statistical analyses of all the metabolite species identified across all samples were conducted. 2D-PCA scores plots of the aforementioned intra- and extracellular metabolic profiles (Figure 2A,B, respectively; see Supplementary Figure S7 for corresponding PCA loadings plots; see Supplementary Files S1 and S2 for complementary PCA loadings values) demonstrated that MΦs exposed to PCM, BCM, and control media were metabolically distinct from each other, with the most notable separations occurring between control MΦs and PCM-/BCM-exposed MΦs along the principal component 1 (PC1) dimensions of the 2D-PCA scores plots of the intra- and extracellular metabolic profiles (Figure 2A,B, respectively). These intra- and extracellular metabolite data sets were further subjected to HCA and heat map visualization to assess which metabolites substantially contributed to the discrimination between PCM-exposed, BCM-exposed, and control media MΦs (Figure 2C and 2D, respectively). Each experimental group presented a particular metabolic signature that was not noted in the other MΦ groups. These features included increased intracellular concentrations of metabolites such as glycerol, phenylalanine, and valine in PCM-exposed MΦs, taurine, creatine, and reduced glutathione in BCM-exposed MΦs, and propionate, methionine, and myo-inositol in control MΦs as shown in Figure 2C. Distinct extracellular characteristics included elevated concentrations of metabolites in cell culture media such as serine, mannose, and threonine in PCM-exposed MΦs, glycerol in BCM-exposed MΦs, and arginine, proline, and fructose in control MΦs, relative to sham cell culture media extract controls, as indicated in Figure 2D.

### 3.3. BCM-Exposed MΦs Exhibit Distinct Metabolic Characteristics Relative to PCM-Exposed MΦs

**Figure 3:**
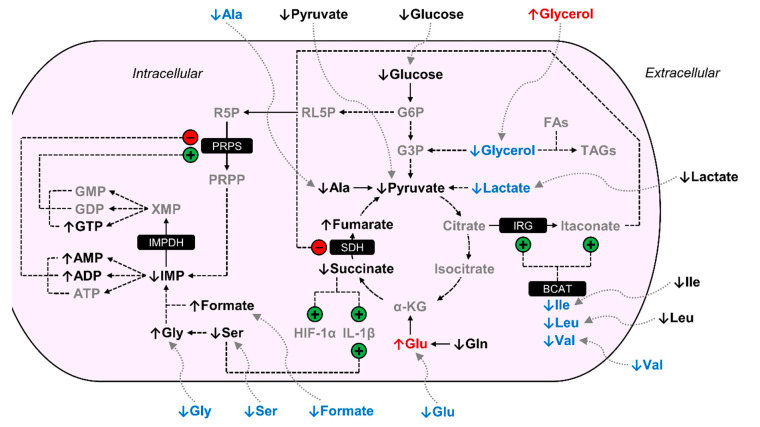
BCM-exposed MΦs are metabolically distinct from PCM-exposed MΦs. Arrows adjacent to metabolite names are indicative of the metabolite level in BCM-exposed MΦs relative to PCM-exposed MΦs. Metabolites in gray were not detected in our NMR spectra. Metabolites in black were detected but were not significantly increased or decreased in BCM-exposed MΦs relative to PCM-exposed MΦs. Metabolites in red were significantly elevated in BCM-exposed MΦs relative to PCM-exposed MΦs, and metabolites in blue were significantly reduced in BCM-exposed MΦs relative to PCM-exposed MΦs. Regulation demonstrated by previous studies that are mentioned in the discussion are indicated with circles containing either a +/green for stimulation or -/red for inhibition. Abbreviations denote: ADP, adenosine diphosphate; Ala, alanine; AMP, adenosine monophosphate; ATP, adenosine triphosphate; BCAT, branched-chain aminotransferase; FAs, fatty acids; G3P, glyceraldehyde 3-phosphate; G6P, glucose 6-phosphate; GDP, guanosine diphosphate; Gln, glutamine; Glu, glutamate; Gly, glycine; GMP, guanosine monophosphate; GTP, guanosine triphosphate; HIF-1α, hypoxia-inducible factor-1α; IL-1β, interleukin-1β; IMP, inosine monophosphate; IMPDH, IMP dehydrogenase; IRG, immune-responsive gene; Ile, isoleucine; Leu, leucine; PRPP, 5-phosphoribosyl-1-pyrophosphate; PRPS, PRPP synthetase; R5P, ribose 5-phosphate; RL5P, ribulose 5-phosphate; SDH, succinate dehydrogenase; Ser, serine; TAGs, triacylglycerides; Val, valine; XMP, xanthosine monophosphate; α-KG, α-ketoglutarate.

BCM-exposed MΦs displayed several distinct metabolic trends relative to PCM-exposed MΦs. Intracellular levels of asparagine, glycerol, isoleucine, lactate, leucine, pyroglutamate, tyrosine, and valine were significantly decreased in BCM-exposed MΦs, −1.41, −7.86, −1.38, −1.42, −1.39, −1.32, −1.30, and −1.62-fold, respectively, relative to PCM-exposed MΦs (Figure 3; Table 1). In addition, BCM-exposed MΦs displayed significantly increased intracellular levels of choline and glutamate, 2.00 and 1.25-fold, respectively, relative to PCM-exposed MΦs (Figure 3; Table 1). Extracellular levels of 3-hydroxyisobutyrate, 4-hydroxyproline, acetate, alanine, formate, glutamate, glycine, mannose, methionine, phenylalanine, serine, threonine, and valine were significantly diminished in BCM-exposed MΦ cultures compared to PCM-exposed MΦ cultures, with BCM-exposed MΦs presenting average deficits of −20.64, −534.62, −302.70, −829.65, −236.62, −954.86, −844.93, −95.68, −367.03, −371.54, −1468.66, −484.36, and −1446.36 nmol/mg protein, respectively, and PCM-exposed MΦs exhibiting average concentrations of 0.80, −215.88, −34.91, −158.21, −39.01, −344.05, −75.85, −40.48, −141.62, −139.58, −380.27, 373.43, and −604.31 nmol/mg protein, respectively, relative to sham extracellular extract controls (Figure 3; Table 2). Moreover, BCM-exposed MΦ cultures demonstrated significantly elevated levels of extracellular 3-hydroxybutyrate, glycerol, and o-phosphocholine compared to PCM-exposed MΦ cultures, with BCM-exposed MΦs displaying average concentrations of −21.07, 125.28, and −19.47 nmol/mg protein, respectively, and PCM-exposed MΦs featuring average deficits of −65.98, −1265.91, and −92.94 nmol/mg protein, respectively, relative to sham extracellular extract controls (Figure 3; Table 2).

### 3.4. Disparate Metabolic Patterns are Presented by PCM- and BCM-Exposed MΦs Compared to Control MΦs

MΦs exposed to PCM and BCM exhibited unique metabolic responses relative to control MΦs. PCM-exposed MΦs contained significantly decreased intracellular levels of choline, fructose, and glutamate, −1.83, −3.18, and −1.30-fold, respectively, relative to control MΦs (Table 1; Figure S8). Intracellular levels of glycerol, o-phosphocholine, and valine were significantly increased in PCM-exposed MΦs, 5.62, 1.38, and 1.43-fold, respectively, relative to control MΦs (Table 1; Figure S8). BCM-exposed MΦs demonstrated significantly diminished intracellular levels of methionine, myo-inositol, proline, propionate, and uridine monophosphate, −1.94, −1.28, −1.59, −1.26, and −1.88-fold, respectively, relative to control MΦs (Table 1; Figure S8). Significant elevation of intracellular levels of taurine, 1.20-fold, in BCM-exposed MΦs relative to control MΦs was also observed (Table 1). PCM-exposed MΦs displayed significantly lower extracellular levels of 3-hydroxyisobutyrate, choline, o-phosphocholine, and pyruvate relative to control MΦs, with PCM-exposed MΦs exhibiting average concentrations of 0.80, −64.97, −92.94, and −747.06 nmol/mg protein, respectively, and control MΦs presenting average concentrations of 6.90, −0.81, −0.23, and −41.17 nmol/mg protein, respectively, relative to sham extracellular extract controls (Table 2; Figure S8). In addition, PCM-exposed MΦs presented significantly increased extracellular levels of mannose and threonine relative to control MΦs, with PCM-exposed MΦs demonstrating average concentrations of −40.48 and 373.43 nmol/mg protein, respectively, and control MΦs featuring average deficits of −99.02 and −66.08 nmol/mg protein, respectively, relative to sham extracellular extract controls (Table 2). BCM-exposed MΦs showed significantly reduced extracellular levels of 3-hydroxyisobutyrate, 4-hydroxyproline, acetate, alanine, arginine, asparagine, formate, glutamate, glycine, methionine, phenylalanine, proline, serine, threonine, and tyrosine relative to control MΦs, with BCM-exposed MΦs indicating average deficits of −20.64, −534.62, −302.70, −829.65, −3106.32, −637.79, −236.62, −954.86, −844.93, −367.03, −371.54, −1373.44, −1468.66, −484.36, and −579.01 nmol/mg protein, respectively, and control MΦs displaying average concentrations of 6.90, −46.62, 25.43, −20.93, −568.00, −226.11, 43.92, −50.04, −82.41, −45.48, −1.22, −155.96, −417.67, −66.08, and −55.89 nmol/mg protein, respectively, relative to sham extracellular extract controls (Table 2; Figure S8).

### 3.5. BCM- and PCM-Exposed MΦs Display Similar Metabolic Responses Compared to Control MΦs

PCM- and BCM-exposed MΦs also presented several similar metabolic trends relative to control MΦs. Intracellular levels of acetate, ADP, AMP, arginine, asparagine, betaine, formate, fumarate, glucose, glutamine, glycine, isoleucine, lactate, pyroglutamate, pyruvate, serine, and succinate were significantly reduced in PCM-exposed MΦs, −1.46, −1.98, −1.38, −1.82, −1.91, −2.45, −2.08, −1.45, −3.34, −2.88, −1.27, −1.55, −2.30, −2.39, −2.04, −1.74, and −2.00-fold, respectively, and BCM-exposed MΦs, −1.53, −1.89, −1.27, −2.62, -2.69, −2.06, −1.73, −1.87, −4.17, −3.57, −1.18, −2.14, −3.27, −3.15, −2.24, −1.93, and −1.92-fold, respectively, relative to control MΦs (Table 1; Figure S8). Furthermore, PCM- and BCM-exposed MΦs displayed significantly increased levels of intracellular IMP, 9.25 and 8.67-fold, respectively, relative to control MΦs (Table 1; Figure S8). Extracellular levels of 3-hydroxybutyrate, aspartate, creatine, fructose, glucose, histidine, isoleucine, lactate, leucine, lysine, myo-inositol, pyroglutamate, urea, and valine were significantly decreased in PCM- and BCM-exposed MΦ cultures compared to control MΦ cultures, with PCM-exposed MΦs presenting average deficits of −65.98, −635.40, −81.20, −2232.88, −23845.20, −170.28, −950.15, −886.91, −1059.98, −256.78, −773.68, −2993.47, −3410.97, and −604.31 nmol/mg protein, respectively, BCM-exposed MΦs exhibiting average deficits of −21.07, −1589.74, −71.00, −2737.16, −32361.26, −290.03, −1331.88, −1869.35, −2042.80, −858.23, −916.76, −3236.09, −2291.28, and -1446.36 nmol/mg protein, respectively, and control MΦs displaying average concentrations of 7.94, −249.34, −7.32, −890.66, −7480.88, 13.29, −128.19, 1487.13, −84.52, 2.98, −187.26, −563.35, 43.01, and −69.95 nmol/mg protein, respectively, relative to sham extracellular extract controls (Table 2; Figure S8).

## 4. Discussion

Although previous studies have demonstrated that bacterial colonization of chronic wounds results primarily in biofilm formation [18], which has been correlated with delayed wound healing and chronicity [20], disparities between the interaction of host innate immune cells with planktonic versus biofilm bacteria that may support pathogenesis have yet to be clearly understood. Identification of impaired immunometabolic responses in MΦs upon biofilm challenge will facilitate the development and implementation of novel therapeutics to rescue dysfunctional innate immune cell behaviors and resolve chronic inflammation. In this study, we sought to investigate the immunometabolic impact of *P. aeruginosa*, a prevalent human pathogen found in chronic wounds, PCM and BCM exposure on primary human monocyte-derived MΦs. We demonstrated that BCM-exposed MΦs exhibit distinct metabolic trends with respect to glycerol metabolism, lactate catabolism, and amino acid metabolism, compared to PCM-exposed MΦs. We also determined that PCM- and BCM-exposed MΦs display several common metabolic patterns regarding glycolysis, purine biosynthesis, and myo-inositol metabolism. Although PCM- and BCM-exposed MΦs share several common metabolic responses, our findings collectively indicate that BCM induces a hyperinflammatory metabolic profile in human MΦs relative to PCM, which potentially contributes to MΦ dysfunction during interaction with bacterial biofilms.

We demonstrated that BCM-exposed MΦs exhibit distinct metabolic profiles compared to PCM-exposed MΦs, including glycerol metabolism. Our metabolic data indicated that BCM-exposed MΦs have significantly lower intracellular levels of glycerol (Table 1) and do not consume significant amounts of glycerol from the cell culture media relative to PCM-exposed MΦs (Figure 3; Table 2). Previous studies have shown that glycerol metabolism is important in bacterial pathogenesis [51,52], biofilm formation [53,54], and that biofilm bacteria are more likely to utilize glycerol than planktonic bacteria [55]. For instance, Sun et al. determined that blocking glycerol degradation in *P. aeruginosa* leads to reduced replicative fitness in a murine lung infection model [52]. In addition, glycerol enhances biofilm formation by both *P. aeruginosa* wound and cystic fibrosis isolate strains, PAO1 and FRD1, respectively, when compared to glucose and succinate [54]. Our data suggest that extracellular glycerol is consumed by *P. aeruginosa* biofilms, whereas *P. aeruginosa* planktonic cells do not consume extracellular glycerol. Thus, BCM-exposed MΦs are unable to consume extracellular glycerol in order to potentially support pro-inflammatory metabolic pathways, such as glycolysis and fatty acid synthesis [56,57,58,59] in a manner similar to PCM-exposed MΦs.

In addition, our data indicated that BCM-exposed MΦs exhibit significantly decreased levels of intracellular lactate, relative to PCM-exposed MΦs (Figure 3; Table 1). This finding differs from what was previously demonstrated [39], where a significant increase in both intra- and extracellular lactate was observed in MΦs activated with a combination of lipopolysaccharide (LPS) and interferon-γ relative to resting MΦs. However, MΦs in our current study were only stimulated with bacterial-conditioned media for 1 h prior to metabolite extraction, whereas MΦs in our prior study were activated with purified recombinant human cytokines over a period of 72 h, which may account for this difference. Contrary to lactate dehydrogenase A, lactate dehydrogenase B (LDHB) displays a higher affinity for lactate and preferentially converts this substrate to pyruvate, with concurrent generation of reduced nicotinamide adenine dinucleotide from nicotinamide adenine dinucleotide [60]. In addition, LDHB has been demonstrated to be a key regulatory element of lactate catabolism in oxygenated subpopulations of tumor cells, known as oxidative cancer cells, which prefer to utilize lactate to fuel oxidative metabolism in order to spare glucose for anaerobic, glycolytic tumor cells [61,62]. Furthermore, Brisson et al. determined that LDHB controls basal autophagic flux and lysosomal activity in oxidative cancer cells [63]. Autophagy, a process induced in MΦs by pathogen-associated molecular patterns and pathogen-induced damage associated molecular patterns from bacteria, is known to enable the degradation of intracellular pathogens and participate in the modulation of innate and adaptive immune responses [64,65,66]. We thus speculate that BCM-exposed MΦs convert a significantly greater amount of intracellular lactate to pyruvate in an effort to support recruitment of autophagic machinery following exposure to bacterial-conditioned medium. However, additional studies are needed to further evaluate and validate this metabolic phenomenon.

We also discovered that BCM-exposed MΦs display a discrete signature with regard to amino acid metabolism compared to PCM-exposed MΦs. BCM-exposed MΦs exhibited significantly reduced intracellular levels of branched-chain amino acids, including isoleucine, leucine, and valine, relative to PCM-exposed MΦs (Table 1). In addition, consumption of valine from the cell culture media by BCM-exposed MΦs was significantly elevated, relative to PCM-exposed MΦs (Figure 3; Table 2). Prior work conducted by Papathanassiu et al. established that selective inhibition of branched-chain aminotransferase activity suppressed several metabolic hallmarks of M1 MΦ activation, such as enhanced glycolysis, immune-responsive gene protein levels, and itaconate production [67]. Although we did not detect itaconate, an antimicrobial metabolite known to inhibit succinate dehydrogenase [35,68,69], in any of our MΦ cell cultures; previous studies have determined that itaconate accumulation begins after 8–10 h of LPS stimulation in RAW264.7 cells [70] and primary murine MΦs [35]. Therefore, we attribute the absence of itaconate signals in our NMR spectra to the fact that we only investigated an early, 1 h, BCM and PCM exposure time point in our current study.

BCM-exposed MΦs also exhibited significantly increased intracellular levels of glutamate (Table 1) and consumed a significantly greater amount of alanine, formate, glutamate, glycine, and serine from the cell culture media, relative to PCM-exposed MΦs (Figure 3; Table 2). Glutamate anaplerosis, a process that replenishes tricarboxylic acid cycle intermediates, and subsequent entry into the tricarboxylic acid cycle as α-ketoglutarate, is known to support the synthesis of succinate [34,71], which is critical for the stabilization of hypoxia-inducible factor-1α and induction of interleukin-1β in M1 MΦs [34,72]. Alanine, which can undergo transamination to pyruvate, has been shown to increase intracellularly in MΦs following LPS stimulation [72]. While we did not observe a concurrent increase in intracellular alanine in BCM-exposed MΦs relative to PCM-exposed MΦs, we speculate that significantly increased consumption of extracellular alanine may be used to maintain basal pyruvate levels in BCM-exposed MΦs. Recent studies have also demonstrated that serine-dependent glycine generation and folate one-carbon metabolism are necessary for interleukin-1β production and purine nucleotide biosynthesis in LPS-stimulated MΦs [73]. Collectively, these findings support that BCM-exposed MΦs elevate their amino acid catabolic capabilities to create a hyperinflammatory metabolic state relative to PCM-exposed MΦs, as has been postulated in previous studies [74,75].

While BCM-exposed MΦs did exhibit several distinct metabolic trends compared to PCM-exposed MΦs, these two MΦ exposure groups also shared common metabolic responses relative to control MΦs, including significantly increased consumption of glucose and fructose from the cell culture media (Table 2) and significantly reduced levels of intracellular glucose and pyruvate (Table 1; Figure S8). Increased glycolytic flux in the presence of oxygen, otherwise known as aerobic glycolysis, is considered to be a universal metabolic characteristic of M1 MΦs [56]. This pathway has been shown to be critical for the regulation and support of M1 MΦ functions, such as the generation of reactive oxygen species, phagocytosis of pathogens and apoptotic cells, and the secretion of pro-inflammatory cytokines [56,57,76]. Furthermore, Cheng et al. demonstrated that leukocytes isolated from patients with sepsis exhibit a shift to aerobic glycolysis, which dissipates following patient recovery [77]. Our findings provide further evidence that pro-inflammatory stimulation of MΦs induces Warburg-like metabolic traits, as previously reported in M1 MΦs [39,57,78]. Accordingly, we postulate that PCM- and BCM-exposed MΦs upregulate glycolysis, a well-known and established metabolic marker of M1 MΦ activation, relative to control MΦs in order to promote M1-like MΦ effector functions.

Furthermore, PCM- and BCM-exposed MΦs displayed significantly increased intracellular levels of IMP, which was accompanied by significantly decreased intracellular levels of serine, glycine, formate, AMP, and ADP, all of which are involved in purine biosynthesis (Table 1; Figure S8). Prior studies have demonstrated that activated human immune cells exhibit increased rates of de novo purine synthesis [79,80] and require sufficient levels of guanosine nucleotides to respond to antigenic and mitogenic stimuli [81]. Keppeke et al. reported that intracellular accumulation of IMP upregulates IMP dehydrogenase (IMPDH) activity by promoting the formation of IMPDH aggregates when GTP production cannot meet cellular demand with non-polymerized IMPDH [82]. Besides IMP, the adequate production of 5-phosphoribosyl-1-pyrophosphate (PRPP) is also essential for purine nucleotide synthesis [81]. PRPP is synthesized from ribose-5-phosphate and adenosine triphosphate by PRPP synthetase, which is inhibited by adenosine nucleotides, AMP and ADP, and stimulated by guanosine nucleotides, guanosine monophosphate, guanosine diphosphate, and GTP (Figure S8) [83]. Our metabolic data support these observations and collectively indicate that PCM and BCM-exposed MΦs may upregulate de novo guanosine nucleotide synthesis in an effort to sustain their guanosine nucleotide pools for effector functions, including the synthesis of tetrahydrobiopterin, which is a co-factor for inducible nitric oxide synthase function [81].

Our data also indicate that PCM- and BCM-exposed MΦs consumed a significantly greater amount of myo-inositol from the cell culture media relative to control MΦs (Table 2); however, only BCM-exposed MΦs exhibited significantly reduced levels of intracellular myo-inositol relative to control MΦs (Table 1; Supplementary Figure S8). Previous studies have suggested that myo-inositol is involved in the regulation of innate immune function [84,85]. Metabolism of myo-inositol leads to the generation of the signaling molecules phosphatidylinositol-3,4,5-trisphosphate (PtdIns(3,4,5)P_3_) and inositol 1,3,4,5-tetrakisphosphate (Ins(1,3,4,5)P_4_). While the latter product, Ins(1,3,4,5)P_4_, suppresses the function of pleckstrin homology (PH) domain-containing protein activity, such as Akt serine/threonine kinase 1, the former product, PtdIns(3,4,5)P_3_, binds to PH domain-containing proteins (Supplementary Figure S8) and as a result activates downstream cellular signaling pathways, which modulate various cellular functions, including the regulation of membrane potential, phagocytosis, and superoxide species production [84,85,86]. Marshall et al. determined that pseudopodia extension and phagocytic particle engulfment was dependent upon type I phosphoinositide 3-kinase (PI3K) activity and the formation of PtdIns(3,4,5)P_3_, which rapidly accumulates at the site of phagocytosis and ensures membrane delivery to distending pseudopods [87]. Moreover, p47^Phox^, an essential subunit of reduced nicotinamide adenine dinucleotide phosphate oxidase, has been shown to be recruited to type I PI3K signals [88]. We therefore hypothesize, within this context, that both PCM- and BCM-exposed MΦs may have a significantly increased demand for myo-inositol and upregulate inositol phosphate metabolism, relative to control MΦs, to potentially modulate phagocytosis and superoxide production.

## 5. Conclusions

In conclusion, our NMR-based metabolic data obtained on primary human monocyte-derived MΦs exposed to *P. aeruginosa* PCM and BCM in vitro have demonstrated that BCM-exposed MΦs possess unique metabolic characteristics when compared to PCM-exposed MΦs, including suppressed glycerol metabolism, increased lactate catabolism, and elevated amino acid metabolism, which may be indicative of a hyperinflammatory metabolic state. Furthermore, we have observed several immunometabolic response trends shared by PCM- and BCM-exposed MΦs compared to control MΦs, including elevated consumption of glycolytic substrates, increased purine biosynthesis, and enhanced inositol phosphate metabolism. These findings emphasize the importance of elucidating the immunometabolic modulatory differences between host cell interactions with planktonic versus biofilm bacteria. Further characterization of these types of interactions may aid in the development of novel therapeutics to rescue dysfunctional hyperinflammatory MΦ behaviors and resolve chronic inflammation in wounds that are often colonized by *P. aeruginosa* bacterial biofilms, including venous leg ulcers.

## Figures and Tables

**Table 1:** Discriminatory metabolites in intracellular extracts associated with 1 h PCM and BCM exposure ^1^.

Metabolite	PCM MΦs ^2^		BCM MΦs ^2^		BCM vs. PCM MΦs	
	FC	*p*-Value	FC	*p*-Value	FC	*p*-Value
Acetate	−1.46	*	−1.53	*	−1.05	NS
ADP	−1.98	**	−1.89	**	1.05	NS
AMP	−1.38	*	−1.27	*	1.09	NS
Arginine	−1.82	**	−2.62	**	−1.44	NS
Asparagine	−1.91	**	−2.69	**	−1.41	**
Betaine	−2.45	*	−2.06	*	1.19	NS
Choline	−1.83	*	1.09	NS	2.00	**
Formate	−2.08	*	−1.73	*	1.21	NS
Fructose	−3.18	*	−2.89	NS	1.10	NS
Fumarate	−1.45	*	−1.87	**	−1.28	NS
Glucose	−3.34	**	−4.17	**	−1.25	NS
Glutamate	−1.30	*	−1.04	NS	1.25	*
Glutamine	−2.88	*	−3.57	*	−1.24	NS
Glycerol	5.62	*	−1.40	NS	−7.86	*
Glycine	−1.27	*	−1.18	**	1.08	NS
IMP	9.25 ^3^	**	8.67 ^3^	***	−1.07	NS
Isoleucine	−1.55	*	−2.14	*	−1.38	*
Lactate	−2.30	*	−3.27	*	−1.42	*
Leucine	−1.04	NS	−1.45	NS	−1.39	*
Methionine	−1.26	NS	−1.94	*	−1.54	NS
myo-Inositol	−1.11	NS	−1.28	*	−1.15	NS
*O*-Phosphocholine	1.38	*	1.29	NS	−1.07	NS
Proline	−1.42	NS	−1.59	*	−1.12	NS
Propionate	−1.19	NS	−1.26	*	−1.06	NS
Pyroglutamate	−2.39	*	−3.15	*	−1.32	**
Pyruvate	−2.04	*	−2.24	*	−1.10	NS
Serine	−1.74	*	−1.93	**	−1.11	NS
Succinate	−2.00	*	−1.92	*	1.05	NS
Taurine	−1.11	NS	1.20	*	1.34	NS
Tyrosine	−1.07	NS	−1.39	NS	−1.30	*
UMP	−1.45	NS	−1.88	**	−1.30	NS
Valine	1.43	*	−1.13	NS	−1.62	*

^1^ Discriminatory metabolites were chosen based upon fold change (FC) of intracellular metabolite concentrations (nmol/mg protein) obtained from spectral fitting patterns using Chenomx NMR Suite software and statistical significance. FC increases are reported as positive values, and FC decreases are reported as negative values. Statistical significance was measured using unpaired parametric two-tailed *t*-tests with Welch’s correction, whereby *, *p* < 0.05; **, *p* < 0.01; ***, *p* < 0.001. Abbreviations denote: NS, not significant; ADP, adenosine diphosphate; AMP, adenosine monophosphate; IMP, inosine monophosphate; UMP, uridine monophosphate. ^2^ Relative to control MΦs. ^3^ FC was calculated using limit of detection (LOD) value (see Table S4) for control MΦ group.

**Table 2:** Discriminatory metabolites in extracellular extracts associated with 1 h PCM and BCM exposure ^1^.

Metabolite	Concentration (mean ± SD)			*p*-value		
	Control MΦs	PCM MΦs	BCM MΦs	PCM ^2^	BCM ^2^	BCM vs. PCM
3-Hydroxybutyrate	7.94 ± 8.71	−65.98 ± 2.54	−21.07 ± 2.44	**	*	****
3-Hydroxyisobutyrate	6.90 ± 2.29	0.80 ± 2.41	−20.64 ± 6.81	*	*	*
4-Hydroxyproline	−46.62 ± 26.71	−215.88 ± 104.10	−534.62 ± 143.50	NS	*	*
Acetate	25.43 ± 18.11	−34.91 ± 48.50	−302.70 ± 100.15	NS	*	*
Alanine	−20.93 ± 36.91	−158.21 ± 81.82	−829.65 ± 237.35	NS	*	*
Arginine	−568.00 ± 138.16	−1980.38 ± 613.21	−3106.32 ± 846.80	NS	*	NS
Asparagine	−226.11 ± 114.24	−193.57 ± 243.09	−637.79 ± 183.49	NS	*	NS
Aspartate	−249.34 ± 46.71	−635.40 ± 142.92	−1589.74 ± 483.01	*	*	NS
Choline	−0.81 ± 4.71	−64.97 ± 5.81	−152.90 ± 63.85	***	NS	NS
Creatine	−7.32 ± 12.01	−81.20 ± 29.44	−71.00 ± 19.09	*	*	NS
Formate	43.92 ± 43.80	−39.01 ± 31.66	−236.62 ± 66.66	NS	**	*
Fructose	−890.66 ± 324.03	−2232.88 ± 532.77	−2737.16 ± 825.08	*	*	NS
Glucose	−7480.88 ± 1804.55	−23845.20 ± 4976.92	−32361.26 ± 8719.05	*	*	NS
Glutamate	−50.04 ± 75.38	−344.05 ± 268.89	−954.86 ± 223.92	NS	*	*
Glycerol	−676.16 ± 416.36	−1265.91 ± 398.51	125.28 ± 124.64	NS	NS	*
Glycine	−82.41 ± 74.53	−75.85 ± 133.27	−844.93 ± 248.20	NS	*	*
Histidine	13.29 ± 15.51	−170.28 ± 73.99	−290.03 ± 90.29	*	*	NS
Isoleucine	−128.19 ± 54.32	−950.15 ± 222.44	−1331.88 ± 370.25	*	*	NS
Lactate	1487.13 ± 589.49	−886.91 ± 725.71	−1869.35 ± 839.64	*	**	NS
Leucine	−84.52 ± 21.47	−1059.98 ± 148.64	−2042.80 ± 569.71	**	*	NS
Lysine	2.98 ± 27.87	−256.78 ± 22.48	−858.23 ± 247.75	***	*	NS
Mannose	−99.02 ± 17.95	−40.48 ± 10.90	−95.68 ± 20.40	*	NS	*
Methionine	−45.48 ± 23.01	−141.62 ± 62.69	−367.03 ± 87.79	NS	*	*
myo-Inositol	−187.26 ± 52.51	−773.68 ± 187.85	−916.76 ± 246.42	*	*	NS
*O*-Phosphocholine	−0.23 ± 8.71	−92.94 ± 6.27	−19.47 ± 10.81	***	NS	**
Phenylalanine	−1.22 ± 20.74	−139.58 ± 69.60	−371.54 ± 103.40	NS	*	*
Proline	−155.96 ± 73.49	−705.16 ± 251.50	−1373.44 ± 429.60	NS	*	NS
Pyroglutamate	−563.35 ± 340.29	−2993.47 ± 597.73	−3236.09 ± 925.54	**	*	NS
Pyruvate	−41.17 ± 19.08	−747.06 ± 25.79	−773.20 ± 421.83	****	NS	NS
Serine	−417.67 ± 177.14	−380.27 ± 113.26	−1468.66 ± 441.28	NS	*	*
Threonine	−66.08 ± 61.60	373.43 ± 26.50	−484.36 ± 84.08	**	**	**
Tyrosine	−55.89 ± 53.16	−265.40 ± 141.53	−579.01 ± 150.66	NS	*	NS
Urea	43.01 ± 151.53	−3410.97 ± 1002.71	−2291.28 ± 454.86	*	**	NS
Valine	−69.95 ± 23.42	−604.31 ± 175.38	−1446.36 ± 397.74	*	*	*

^1^ Discriminatory metabolites were chosen based upon statistical significance of extracellular metabolite concentrations (nmol/mg protein) obtained from spectral fitting patterns using Chenomx NMR Suite software. Concentrations of extracellular metabolites were normalized to sham culture media controls, whereby increases are reported as positive values and decreases are reported as negative values. Statistical significance was measured using unpaired parametric two-tailed *t*-tests with Welch’s correction, whereby *, *p* < 0.05; **, *p* < 0.01; ***, *p* < 0.001; ****, *p* < 0.0001. Abbreviations denote: NS, not significant; SD, standard deviation. ^2^ Relative to control MΦs.

## Supplementary Materials

The following are available online at https://www.mdpi.com/2073-4409/9/10/2260/s1, Figure S1: Purity of isolated monocytes; Figure S2: Phenotype of primary human monocyte-derived MΦs; Figure S3: Description of (A) preparation of PCM, BCM, and control exposure media, (B) experimental and sham media control flask setup, and (C) normalization of experimental (spent) media metabolite concentrations using sham media control metabolite profiles; Figure S4: Evaluation of metabolic similarity between different exposure group sham media samples; Figure S5: Photograph of *Pseudomonas aeruginosa* biofilm colonies grown on a tissue culture insert; Figure S6: 1D ^1^H NMR spectra acquired on MSU’s Bruker 600 MHz (^1^H Larmor frequency) NMR spectrometer on extracellular metabolite extracts from MΦs; Figure S7: PCA loadings plot for (A) intra- and (B) extracellular MΦ metabolite extracts; Figure S8: BCM- and PCM-exposed MΦs share common metabolic trends relative to control MΦs; File S1: Intracellular PCA loadings; File S2: Extracellular PCA loadings; Table S1: Comparison of select metabolite concentrations in different exposure group sham media controls; Table S2: Summary of unique metabolite species, by class, identified using 1D ^1^H-NMR; Table S3: Metabolite detection across all samples; Table S4: 1D ^1^H-NMR intra- and extracellular metabolite limit of detection (LOD) values.
